# The Focusing Properties of a Modular All-Metal Lens in the Near-Field Region

**DOI:** 10.3390/s24165092

**Published:** 2024-08-06

**Authors:** Qifei Zhang, Linyan Guo, Yunqing Li, Chen Wang

**Affiliations:** 1 School of Geophysics and Information Technology, China University of Geosciences, Beijing 100083, China; qifeizhang@email.cugb.edu.cn (Q.Z.); yqli@email.cugb.edu.cn (Y.L.); cwang@email.cugb.edu.cn (C.W.); 2 Key Laboratory of Intraplate Volcanoes and Earthquakes, Ministry of Education, China University of Geosciences, Beijing 100083, China

**Keywords:** modular, all-metal lens, near-field focusing, low cost

## Abstract

This article proposes a modular and passive all-metal lens to achieve near-field focusing with adjustable focus. The proposed lens consists of all-metal units with the phase coverage range exceeding 360°, and the arrangement of units is guided by the phase compensation method. Specifically, using the strategy of module unit synthesis, the arrangement of lens units under different focuses can be assembled arbitrarily, which reduces the production costs by 39.3% and improves the freedom of lens design. The simulation and experimental results show that the lens exhibits excellent focusing properties and freely changes the position of the expected focus (0.30 m–0.75 m). Therefore, the modular all-metal lens designed in this article has the characteristics of high transparency and a high degree of freedom, which can provide low-cost and lightweight solutions for various applications in the field of antennas, such as near-field target detection, microwave imaging, biomedicine, and so on.

## 1. Introduction

A near-field focusing (NFF) lens has a wide range of applications such as near-field target detection, radar imaging and biomedical applications [1]. In the radar imaging process based on an NFF lens, the electromagnetic waves from different targets produce different signals when they reach the receiving antenna through a converging lens, forming an image of the target [2]. In medical treatment, NFF lenses can focus electromagnetic beams, which have high directionality, thermal efficiency, and microwave thermal gain [3]. However, the traditional lens often suffers from some issues such as its large volume and high cost. Therefore, metamaterial lenses with low profiles, compact structures, and low costs have been widely studied [4,5]. The metamaterial lens focusing system typically consists of a feed source and a metamaterial lens, which is composed of multiple units with different transmission coefficients. Lens units are distributed according to the specific phase correspondence and converge electromagnetic waves in the near-field of the lens. However, existing metamaterial lenses are generally processed using printed circuit board (PCB) technology based on dielectric substrates, which cannot work in high temperature, high pressure and high mechanical strength. For example, in the aerospace industry, equipment typically needs to be used at temperatures of several hundred degrees. In this case, the metamaterial substrate will deform and melt. Therefore, studying the design method of an all-metal NFF lens with high-temperature resistance and mechanical stability based on metamaterial technology is of great significance [6,7,8,9].

At present, an NFF lens is mainly concentrated in the X band or above [10,11,12,13] because when the operating frequency is low, the physical size of the lens unit will be very large, which leads to a larger overall size of the lens and a decrease in the number of units, resulting in poor focusing performance. Therefore, it is necessary to study small-sized and lightweight all-metal NFF lenses [14,15]. This paper conducts a current analysis based on the Jerusalem groove structure to increase the transparency of the structure and reduce the weight of the lens [16].

**Figure 1:**
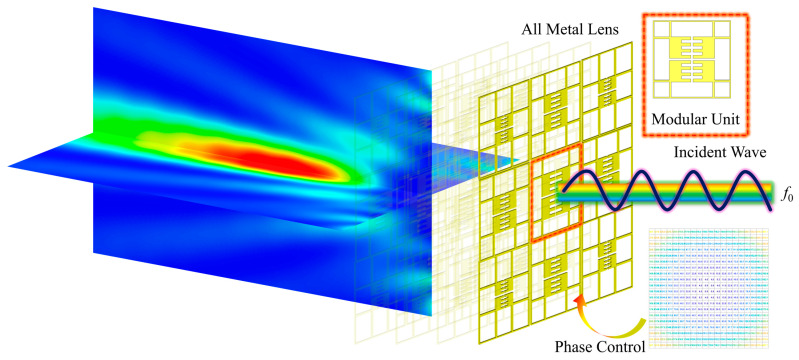
Schematic diagram of near-field focusing with modular all-metal lens.

At present, in the manufacturing of existing all-metal lenses, most research adopts a one-time processing method (such as whole-block slicing, 3D printing, etc.), which makes the lens unit correspond with the phase distribution and cannot dynamically adjust the performance of the fabricated lens [6,14,16]. Specifically, due to the current conduction characteristics of all-metal structures, the traditional PIN-based dynamic adjustment method for adjustable metasurface lenses cannot be achieved in all-metal lenses [17]. In 2021, Andrea Massa proposed the concept of modular electromagnetic skin, which breaks down lenses into modular units to achieve the optimal layout of building electromagnetic shells [18]. Inspired by this concept, this article adopts the modular method to assemble lenses to achieve a free combination of phases, adjustable focus and low manufacturing costs. The difference with electromagnetic skin is that this article introduces the concept of a discrete phase and uses the phase quantization (PQ) method to classify lens units, further reducing the complexity of modular all-metal lens design. The schematic diagram of the structure and focusing effect used in this article is shown in Figure 1.

The remainder of this paper is structured as follows. In Section 2, we present the design process of the lens, explain the focusing method, and provide the simulation results. Section 3 introduces the experimental results of the model. Finally, the conclusion is drawn in Section 4.

## 2. Lens Design

### 2.1. Unit Current Analysis

**Figure 2:**
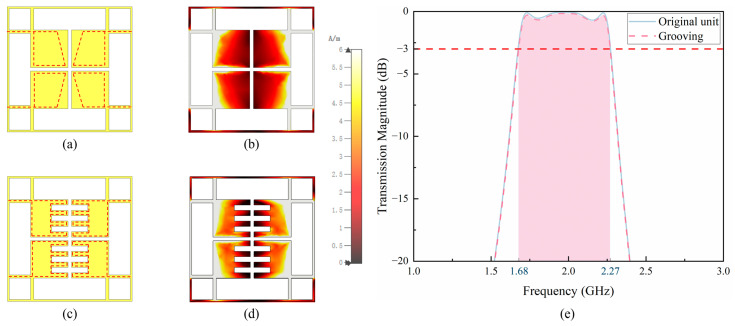
Jerusalem slot unit and its current analysis. (**a**) Lens unit. (**b**) Current distribution of lens unit. (**c**) Grooving lens unit. (**d**) Current distribution of grooving lens unit. (**e**) Transmission amplitude of unit before and after grooving.

According to the completed work [16,19], when an all-metal lens unit is designed in the shape shown in Figure 2a, it can have a good control effect on the phase of electromagnetic waves. However, the transparency of the unit is small, and the structure is thick. The large amount of metal materials makes the lens very heavy, and the production cost is high, making it impossible to mass manufacture it.

In order to reduce the weight of the lens, this study adopts the method of current analysis, which can reduce the weight of the lens unit without changing its radiation performance. Figure 2 shows the Jerusalem slot unit and its current analysis. As shown in Figure 2b, when the lens works at 2.0 GHz, due to the strips surrounding the central patches, the current response of the four-square sections in the middle is relatively small, and they hardly participate in electromagnetic wave radiation near the center. The current path is shown by the red dashed line in Figure 2a,c. The part with a smaller current response in the middle square is grooved, and the unit current distribution after grooving is shown in Figure 2d. Figure 2e shows the transmission amplitude of the unit before and after etching, where the shaded area represents the 3dB working bandwidth of the transmission unit. Since the main participating part of electromagnetic wave radiation has not been changed, the radiation performance of the unit is almost the same as before etching. At this point, the transparency of the unit increased from 58.1% to 62.6%, with a relative transparency increase of 10.7%. The thickness of the grooved unit was 0.6 mm, only 20% of the original unit thickness, which significantly reduced the weight of the lens. The weight of the grooved lens was only 17.9% of the original weight, a decrease of 82.1%.

**Figure 3:**
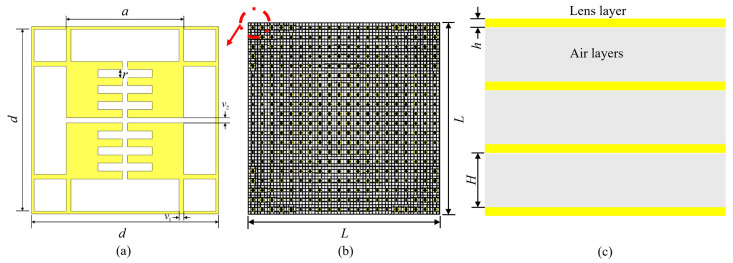
Lens structure. (**a**) Lens unit. (**b**) Top view of overall lens structure. (**c**) Side view of overall lens structure.

The lens structure is shown in Figure 3. The overall framework of the unit is a d×d square structure. To achieve 360° coverage of lens phase, the lens is designed with four layers, each consisting of 20×20 units, with a spacing of *H* between the layers and a thickness of *h*. In fact, when the thickness of the air layer varies between 30 mm and 45 mm, its transmission amplitude is above −3 dB at 2.0 GHz, and the bandwidth is ~480 MHz. When the thickness is too high or too low, the transmission amplitude will decrease significantly at some frequencies. Therefore, this article chooses 37.5 mm as the air layer thickness after considering the operating frequency and bandwidth. So, the total thickness is 4×h+3×H= 114.9 mm. By adjusting the length of the current path, *a*, the amplitude and phase of the lens unit can be adjusted accordingly. The specific size parameters of the lens are shown in Table 1. Therefore, for the designed lens, its near-field boundary should be $2D^{2}/\lambda =2\times {\left(\sqrt{2}L\right)}^{2}/\lambda =13.82\;m$, wherein *D* is the aperture size of the lens.

**Figure 4:**
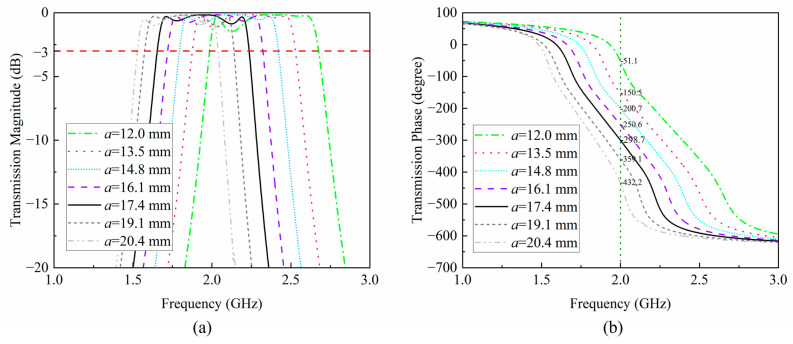
Unit transmission amplitude and phase under different values of *a.* (**a**) Amplitude distribution. (**b**) Phase distribution.

Research has shown that the edge length, *a*, of the metal within the unit can affect the amplitude and phase of transmission [16]. In order to investigate the influence of *a* on the transmission performance of the unit after grooving, this paper analyzes the variation range of the transmission amplitude and phase of the unit when *a* is different, as shown in Figure 4a,b. As shown in the figure, when *a* varies between 12.0 mm and 20.4 mm, the transmission amplitude of the unit at 2.0 GHz is greater than −3 dB, demonstrating good transmission performance. The transmission phase varies between −51.1° and −432.2°, with a difference of 381.1°. Therefore, adjusting the length of the current path can enable the lens unit to achieve a phase change of over 360° while ensuring transmission efficiency. Therefore, this unit can be used to design the NFF lens based on phase modulation.

**Figure 5:**
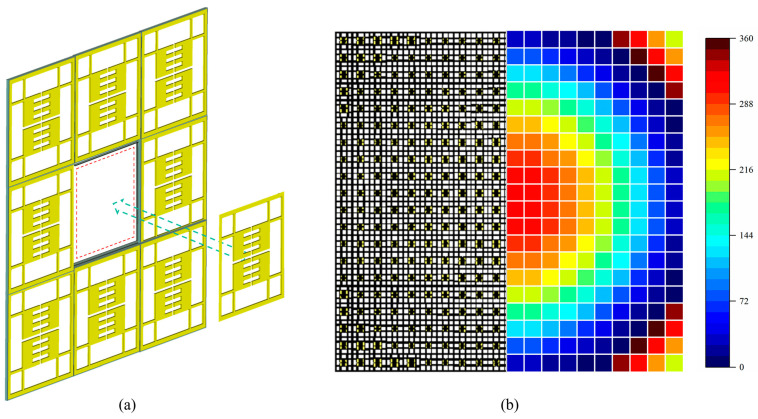
(**a**) Schematic diagram of modular lens units synthesized. (**b**) Lens and corresponding phase distribution.

A traditional lens is produced using the whole-block processing method [20,21], which results in high production costs and the inability to change the focus of the lens. Therefore, this study adopts a modular design approach, where lens units with different phases are processed using a single-module structure. The lens array is assembled using the method of building blocks to achieve different phase combinations. When the focus changes, it is simple to assemble according to the calculated phase correspondence to allow electromagnetic waves to converge at different expected focuses, greatly reducing production costs. Taking the experiment in this article as an example, when the expected focus is 0.3 m, 0.5 m, and 0.75 m, respectively, the required number of lens units is reduced from 4800 (i.e., 20 × 20 units/layer × four layers × three focuses) to 2912, a decrease of 39.3%. From the theoretical analysis, due to the reusability of lens units, the more times the expected focus is adjusted, the lower the cost of the modular design method proposed in this study compared to traditional processes. Figure 5a is a schematic diagram of the block method for splicing lenses, with the blue part representing the framework of the supporting lens unit. Acrylic material is used to reduce the interference of the supporting structure on electromagnetic waves. Figure 5b is a schematic diagram of the lens and its corresponding phase distribution, consisting of two parts, wherein the left half is the splicing result of the lens unit, and the right half is the corresponding phase distribution. That is, each lens unit corresponds to a unique phase to achieve the near-field focusing.

### 2.2. Near-Field Energy Focusing

**Figure 6:**
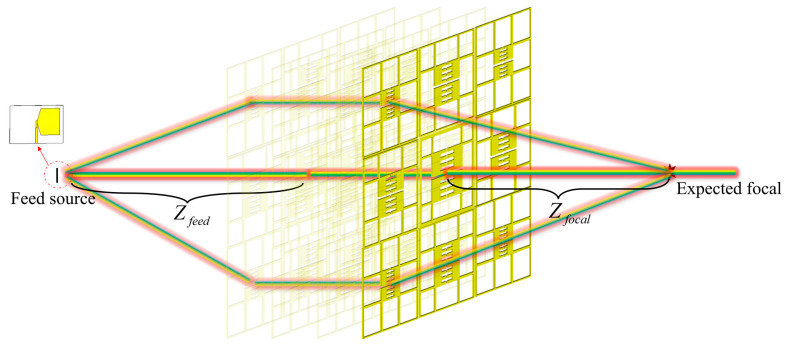
Theoretical principle of near-field energy focusing.

This study designs an NFF lens based on the phase compensation algorithm [22], and the schematic diagram of the theoretical principle is shown in Figure 6. The phase compensation algorithm achieves the focusing effect by compensating for the distance difference between different transmitting and receiving units and the focus. The required phase for each unit is calculated by using Formula (1), wherein $Z_{feed}$ is the distance between the feed antenna and the lens, $Z_{focal}$ is the distance from the focus to the lens, $r_{i}$ is the distance from any lens unit to the center point of the lens, and $\phi_{0}$ is the original phase of the electromagnetic wave.

Using a butterfly antenna as the feed antenna for the lens, when $Z_{feed}$ is 0.61 m, due to the lens being in the far-field region of the feed antenna, the radiated electromagnetic waves can be considered as plane waves when they reach the lens, which means that $\phi_{0}=0$.

(1) $$\varphi_{i}=\frac{2\pi }{\lambda_{0}}\times (R_{1}+R_{2})+\phi_{0}=\frac{2\pi }{\lambda_{0}}\times (\sqrt{Z_{feed}^{2}+r_{i}^{2}}+\sqrt{Z_{focal}^{2}+r_{i}^{2}})+\phi_{0}$$

**Figure 7:**
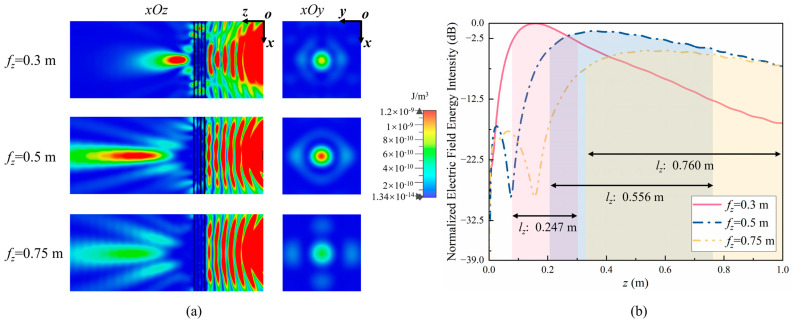
(**a**) Electric field energy in the *yOz* and *xOy* planes. (**b**) Normalized electric field energy intensity along the *z*-axis.

In the fields of near-field work such as radar imaging, medicine, and wireless energy transmission, the target object is generally within 1 m of the lens. Therefore, the expected focuses, $f_{z}$, in this study are 0.3 m, 0.5 m, and 0.75 m, respectively, to study the differences in electromagnetic wave energy at different focal lengths in the near-field work area. Using a butterfly antenna as the feed source, the *xOy* and *xOz* cross-sections of the electric field energy intensity are obtained using the full-wave simulation method, as shown in Figure 7a, wherein electromagnetic energy converges at the expected focus in the *xOy* plane, presenting a clear focus. Figure 7b shows the energy intensity along the *z*-axis in the *xOy* plane. As can be seen from the graph, the speed of energy attenuation slows down with the increase in focal length, and the length of the focus increases accordingly. Using $l_{z}$ to describe the length of the focus in the *z*-axis, when $f_{z}$ is 0.3 m, 0.5 m, and 0.75 m, $l_{z}$ is 0.247 m, 0.556 m, and 0.760 m, respectively. This indicates that as the focal length increases, the electromagnetic wave energy is more evenly distributed around the focus, but the maximum energy at the focus decreases, weakening the energy convergence ability.

**Figure 8:**
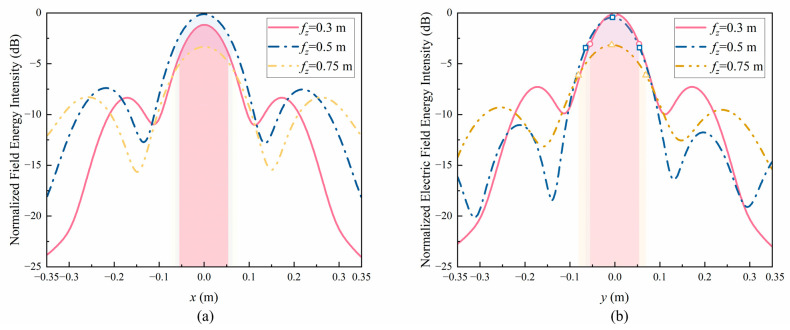
Normalized electric field energy intensity along different coordinate axes: (**a**) *x*-axis, (**b**) *y*-axis.

In order to more accurately determine the size of the focus, the electric field energy intensity along the *x-* and *y*-axes at the focal plane was analyzed as shown in Figure 8a,b. As seen in the graph, as the focal length increases, there is no significant change in $l_{x}$ and $l_{y}$ between 0.1 m and 0.15 m. Therefore, the focus is an elliptical shape that continuously extends in the *z*-axis but does not change much in the *x-* and *y*-axes. The numerical conclusions are shown in columns 2 to 4 of Table 2.

As already known [23], the peak energy density of the radiated electric field does not appear at the expected focusing position, indicating the presence of focal shift. Therefore, the maximum field intensity point was defined as $\eta_{0}$ to study the accuracy of focusing, and the numerical conclusions are shown in column 5 of Table 2. Due to energy attenuation, the value of $\eta_{0}$ is less than $f_{z}$ but still within the 3 dB loss region. $E_{f_{z}}$, $E_{\eta_{0}}$ and $E_{\mathrm{unfocused}}$ respectively represent the electric field intensity at positions $f_{z}$ and $\eta_{0}$ during focusing and at corresponding positions when unfocused. In addition, in order to study the field enhancement effect of the lens at the expected focus, the field enhancement factor, α, is defined as the difference between the electric field intensity at the expected focus, $f_{z}$, and the electric field intensity under unfocused conditions at the same position:

(2) $$\alpha =\left|E_{f_{z}}-E_{\mathrm{unfocused}}\right|$$

In addition, in order to study the true field enhancement effect of the lens, the field enhancement factor β is defined as the difference between the electric field intensity at $\eta_{0}$ and the electric field intensity under unfocused conditions at the same position:

(3) $$\beta =\left|E_{\eta_{0}}-E_{\mathrm{unfocused}}\right|$$

Moreover, in order to study the error caused by focal shift, the error factor, ϑ, is defined as the difference between the electric field intensity at the expected focus, $f_{z}$, and the electric field intensity at the maximum field intensity point, $\eta_{0}$, as follows:

(4) $$\vartheta =\left|E_{\eta_{0}}-E_{f_{z}}\right|$$

The sixth, seventh, and eighth columns of Table 2 provide numerical conclusions for α, β, ϑ and for different expected focuses, $f_{z}$. The maximum field enhancement factor, α, is 4.8 dB, and the maximum field enhancement factor, β, is 5.3 dB, indicating that the lens can significantly enhance the energy of the electric field. In addition, the error factors, ϑ, of the three focusing situations are all less than 1 dB, which means that $f_{z}$ and $\eta_{0}$ are all within the energy focusing region and the lens can achieve precise focusing.

### 2.3. Phase Quantization Analysis

**Figure 9:**
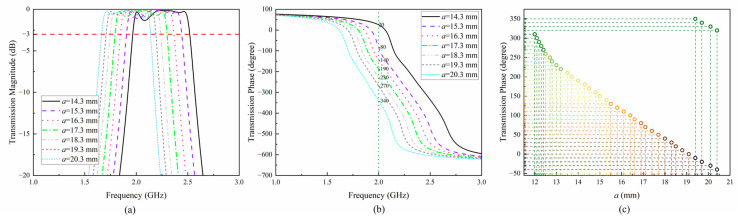
(**a**) Quantized transmission amplitude. (**b**) Quantized transmission phase. (**c**) Corresponding relationship between current path *a* and transmission phase.

In order to further reduce costs, the continuous phase generated by the lens unit is quantified in steps of 10°. Compared to the continuous phase, only 36 different lens units are needed to achieve near-field energy focusing. To verify the feasibility of PQ, the transmission amplitude and phase of the lens unit are derived as shown in Figure 9a,b. The results show that when *a* varies between 14.3 mm and 20.3 mm, the transmission amplitude of the unit at 2.0 GHz is greater than −3 dB, exhibiting good transmission performance. The transmission phase varies between 20° and −340°, and the phase transformation amount reaches 360°. Therefore, the quantified units still meet the basic conditions for designing transmissive lenses. Because the transmission phase of the lens unit changes with the current path length, *a*, the desired phase can be obtained by adjusting the value of *a*. Figure 9c shows the correspondence between the current path, *a,* and the transmission phase. As shown in the figure, when *a* varies between 12.0 mm and 20.4 mm, 36 different values of the quantization phase are taken to achieve 360° full coverage of the phase change.

**Figure 10:**
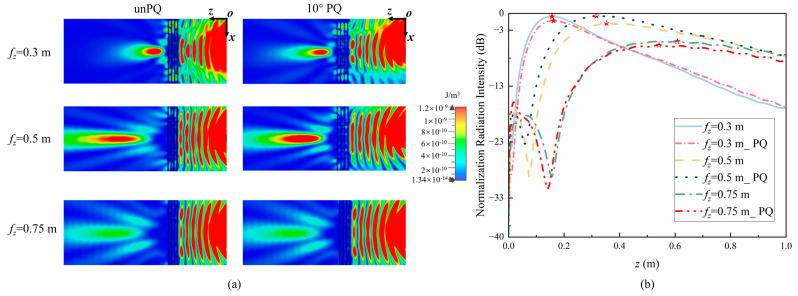
(**a**) Electric field energy of the *xOz* plane when unPQ and PQ. (**b**) Normalized electric field energy intensity along the *z*-axis when unPQ and PQ.

To verify the focusing effect of the quantized lens, the full wave simulation method was used to obtain the electric field energy after PQ in the *xOz* plane, as shown in Figure 10a. It can be seen that there is no significant difference in the energy convergence effect between PQ and unPQ. Figure 10b shows the energy intensity along the *z*-axis in the *xOz* plane with PQ and unPQ. In Figure 10b, the maximum energy value on the curve is marked with pentagrams with different fillings, and the curves before and after quantization match well. Therefore, the quantified lens unit can be used to achieve near-field energy focusing.

To provide a better overview of the contribution of this work and underscore the necessity of miniaturized design and modular methods, Table 3 compares some of the most advanced all-metal designs with this work. As reported in this table, the proposed model reduces the size of the model through current analysis methods and achieves free adjustment in a modular manner at an extremely low cost.

## 3. Experiment

### 3.1. Experimental System Construction

**Figure 11:**
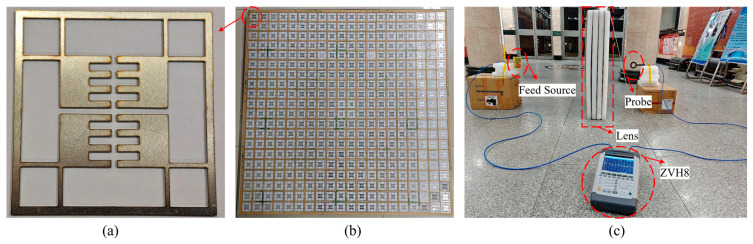
Experimental system setup. (**a**) Lens unit. (**b**) All-metal NFF lens. (**c**) Experimental environment.

To verify the simulation results, the free space method was used to test the lens, and an experimental system was constructed as shown in Figure 11, wherein Figure 11a shows the all-metal lens unit, and Figure 11b shows the overall structure of the assembled metal lens. In order to reduce the impact of the support structure on the lens performance, the modular lens unit is fixed as a whole with acrylic plate and foam filler. Figure 11c shows the experimental environment, where a vector network analyzer (ZVH8) is used as the signal transmitter and receiver, and the feed antenna is a butterfly antenna operating at 1.0–3.0 GHz [24]. The electromagnetic energy probe used in the test is FOSTTEK’s NFP–ONE series probe, which can accurately detect the electromagnetic energy intensity in the DC–20 GHz frequency band. The distance between the feed source and the lens is 0.61 m, and both the feed source and the electromagnetic energy probe are facing the center point of the lens, consistent with the simulation setting.

### 3.2. Results Evaluation

**Figure 12:**
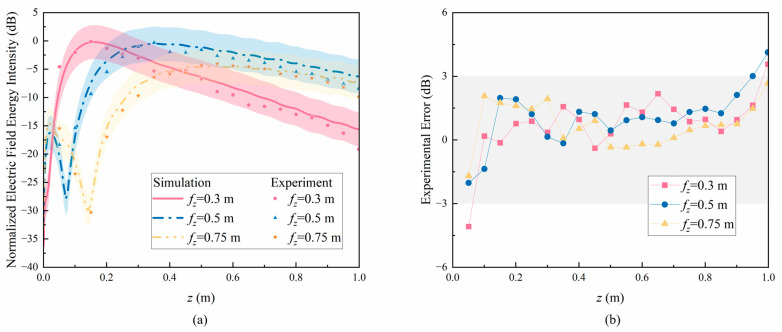
(**a**) Experimental results in the *z*-axis with different $f_{z}$. (**b**) Experimental errors along the *z*-axis.

When the electromagnetic energy probe moves along the *z*-axis, the measured electric field energy intensity is shown, as demonstrated in Figure 12, with 0.05 m between each test point. Moreover, this study conducted an error analysis on the test results to determine their reliability. In Figure 12a, red, blue, and yellow represent the simulation and test results at 0.3 m, 0.5 m, and 0.75 m, respectively. The line graph represents the simulation results, and the scatter plot represents the test results. In order to obtain a more intuitive deviation between the simulation and the experiment results, a 3 dB error area was defined, and shadow areas of the same color were used to represent the difference between the simulation results and ±3 dB. As shown in the figure, most of the experimental results are within the 3 dB error region. Figure 12b shows the experimental error along the *z*-axis, which is the difference between the simulation and the experiment results. When the experiment distance is between 0.1 m and 0.9 m, the experimental errors are all less than 3 dB, indicating that the experiment results are in good agreement with the simulation results, verifying the reliability of the experiment.

**Figure 13:**
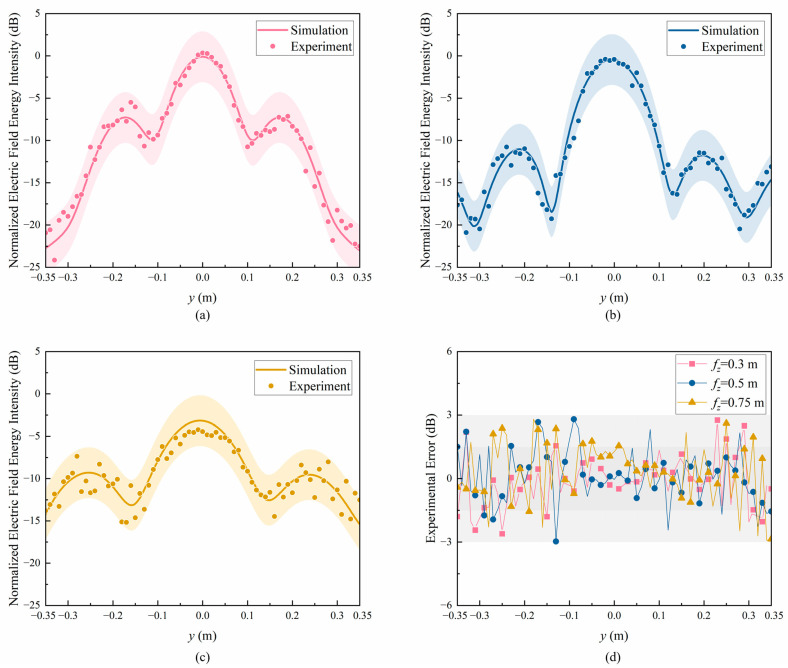
Experimental data and errors along the *y*-axis of the focal plane at different focuses: (**a**) *f_z_* = 0.3 m; (**b**) *f_z_* = 0.5 m; (**c**) *f_z_* = 0.75 m; (**d**) experimental errors.

Figure 12 shows the measured electric field energy intensity when the electromagnetic energy probe moves in the *y*-axis, with 0.01 m between each test point to obtain more accurate results. Figure 13a–c show the results when $f_{z}$ is 0.3 m, 0.5 m, and 0.75 m, respectively. The line graph represents the simulation values, and the scatter plot represents the experiment values. Similarly, in order to obtain more intuitive deviations between the simulation and experiment results, a 3 dB error area is defined, with shaded areas of the same color representing the difference between the simulation results and ±3 dB. It can be seen that when the test point is between −0.1 m and 0.1 m, the experiment results are in good agreement with the simulation results, and the field energy enhancement effect is very significant. When the test point is far from the energy center, due to electromagnetic diffraction phenomena and interference from the experimental environment, the experimental field energy intensity fluctuates to a certain extent, but the experimental errors are all within the 3 dB error range. Figure 13d shows the experimental errors. At 0.3 m, 0.5 m, and 0.75 m, the experimental errors are all within the 3 dB error range. Moreover, when the test points are between −0.1 m and 0.1 m, most of the experimental errors are within the 1.5 dB error range. This indicates that the experiment results are in good agreement with the simulation results, verifying the reliability of the test.

## 4. Conclusions

This paper proposes a modular all-metal lens designed for NFF. Using the novel modular design method, the proposed lens can achieve adjustable focus between 0.30 m and 0.75 m at 2.0 GHz, significantly enhancing the field intensity level at the expected focus, which is about 5 dB higher than when unfocused. This indicates that the proposed lens has good phase modulation and electromagnetic wave control capabilities, effectively converging electromagnetic wave. Compared with the traditional lens, the modular all-metal lens provides low-cost, lightweight, and highly flexible solutions, which have good application prospects in near-field target detection, microwave imaging, biomedical applications and other fields.

## Figures and Tables

**Table 1:** Size parameter of lens (mm).

*a*	*d*	*r*	$v_{1}$	$v_{2}$	*L*	*H*	*h*
20.3	36	1.4	1	0.9	700	37.5	0.6

**Table 2:** Focusing area parameters at different focuses.

$f_{z}\;(m)$	$l_{x}\;(m)$	$l_{y}\;(m)$	$l_{z}\;(m)$	$\eta_{0}\;(m)$	α (dB)	β (dB)	ϑ (dB)
0.30	0.109	0.110	0.247	0.222	4.3	5.2	0.9
0.50	0.120	0.127	0.556	0.352	4.4	5.1	0.7
0.75	0.145	0.150	0.760	0.620	4.8	5.3	0.5

**Table 3:** Comparison with the recent all-metal lenses.

Reference No.	Waveband	Center Frequency (GHz)	Unit Size (mm)	Unit Thickness (mm)	Adjustable
[6]	X	10.30	0.34λ×0.34λ	0.25	No
[7]	Ku	14.25	0.31λ×0.31λ	11.66	Yes
[14]	X	11.50	0.44λ×0.44λ	1	No
[16]	L	1.59	0.19λ×0.19λ	3	No
[19]	L	1.60	0.19λ×0.19λ	2	No
This work	LS	2.00	0.24λ×0.24λ	0.6	Yes

## Data Availability

The original contributions presented in the study are included in the article, further inquiries can be directed to the corresponding author.
